# Peculiarities and Consequences of Different Angiographic Patterns of STEMI Patients Receiving Coronary Angiography Only: Data from a Large Primary PCI Registry

**DOI:** 10.1155/2020/9839281

**Published:** 2020-07-20

**Authors:** Alexandru Burlacu, Grigore Tinica, Bogdan Artene, Paul Simion, Diana Savuc, Adrian Covic

**Affiliations:** ^1^Department of Interventional Cardiology, Cardiovascular Diseases Institute, “Grigore T. Popa” University of Medicine, Iasi, Romania; ^**2**^ Department of Cardiovascular Surgery, Cardiovascular Diseases Institute, “Grigore T. Popa” University of Medicine, Iasi, Romania; ^3^Department of Interventional Cardiology, Cardiovascular Diseases Institute, Iasi, Romania; ^4^Nephrology Clinic, Dialysis and Renal Transplant Center, “C.I. Parhon” University Hospital, Iasi, Romania; ^5^“Grigore T. Popa” University of Medicine, Iasi, Romania; ^6^The Academy of Romanian Scientists (AOSR), Bucharest, Romania

## Abstract

*Background*. Inappropriate cardiac catheterization lab activation together with false-positive angiographies and no-culprit found coronary interventions are now reported as costly to the medical system, influencing STEMI process efficiency. We aimed to analyze data from a high-volume interventional centre (>1000 primary PCIs/year) exploring etiologies and reporting characteristics from all “blank” coronary angiographies in STEMI. Methods. In this retrospective observational single-centre cohort study, we reported two-year data from a primary PCI registry (2035 patients). “Angio-only” cases were assigned to one of these categories: (a) Takotsubo syndrome; (b) coronary embolisation; (c) myocardial infarction with nonobstructive coronary arteries; (d) myocarditis; (e) CABG-referred; (f) normal coronary arteries (mostly diagnostic errors); and (g)others (refusals and death prior angioplasty). Univariate analysis assessed correlations between each category and cardiovascular risk factors. Results. 412 STEMI patients received coronary angiography “only,” accounting for 20.2% of cath lab activations. Barely 77 patients had diagnostic errors (3.8% from all patients) implying false-activations. 40% of “angio-only” patients (n = 165) were referred to surgery due to severe atherosclerosis or mechanical complications. Patients with diagnostic errors and normal arteries displayed strong correlations with all cardiovascular risk factors. Probably, numerous risk factors “convinced” emergency department staff to call for an angio. Conclusions. STEMI network professionals often confront with coronary angiography “only” situations. We propose a classification according to etiologies. Next, STEMI guidelines should include audit recommendations and specific thresholds regarding “angio-only” patients, with specific focus on MINOCA, CABG referrals, and diagnostic errors. These measures will have a double impact: a better management of the patient, and a clearer perception about the usefulness of the investments.

## 1. Introduction

Ever since coronary angiography was introduced in the early management strategy of patients presenting with acute myocardial infarction (MI) (especially ST elevation myocardial infarction, STEMI), it underscored the importance of the atherothrombotic process manifesting as obstructive coronary artery disease (CAD) in more than 90% of the AMI patients [1]. Strategies focusing on maintaining arterial patency were developed, dramatically improving clinical outcomes and lowering both short- and long-term mortality. Nowadays, the European Society of Cardiology Guidelines on STEMI recommends the implementation of primary percutaneous coronary intervention- (PCI-) capable centers which are able to deliver a 24/7 service and to perform primary angioplasty without delay (indication class I, LoE A [2]).

The main issue in organizing and financing a STEMI network is not “why” but “how” [3]. Investing important amounts of money in a medical system that definitely saves lives generated auditing programs targeting an optimization of healthcare strategies [4]. One should have in mind “Stent for Life” initiative in building STEMI systems of care in emerging countries, which sharply improved the delivery and patient access to the lifesaving indications of primary PCI [5].

Nevertheless, the widespread use of coronary interventions in STEMI revealed a new topic that puzzled clinicians: large registries reported a 13% of STEMIs occurring in the absence of obstructive CAD [6–9] (as high as 25% [10]). This significant new group generated important issues (e.g., dealing with the pathogenic mechanism responsible for the myocardial damage, the appropriate management strategy, or the prognostic of these patients) that are yet to be disentangled. Thus, a new concept was coined as MINOCA, defining myocardial infarction with nonobstructive coronary arteries. Nowadays, a growing interest on MINOCA is manifested; some notable research studies address this issue and propose various management strategies [1, 10–13]. At the opposite pole, 5–10 percent of STEMI patients was reported to receive CABG treatment in acute phase (mainly due to severe CAD unsuitable for PCI or postinfarction ventricular septal rupture or mitral valve regurgitation) [14].

Hence, a common (mis)perception of the funding department from a hospital is that STEMI patients receiving “blank” coronary angiography only (e.g., a procedure not followed by a balloon/stent inflation) waste the hospital resources unnecessarily and reflect a poor management of departmental resources. Indeed, a false-activation of the STEMI team [15] or inappropriate cardiac catheterization lab activation [16] together with false-positive angiographies and no-culprit found coronary interventions are now reported as costly to the medical system, influencing STEMI process efficiency [17]. However, from the interventional cardiologist point of view, both clinical entities (MINOCA and CABG-referred STEMI) require the same activation of the catheterization laboratory team even if only an angiography is performed (as the patients present with chest pain, ST elevation on EKG, and/or raised cardiac troponins).

Therefore, in this study, we aim to analyze two-year data from a high-volume single-interventional centre serving for the eastern part of Romania (the only primary PCI facility for at least 7 million inhabitants, with more than 1000 primary PCIs per year) exploring etiologies and reporting characteristics from all blank coronary angiographies in STEMI. We also pursued a detailed approach of characteristics of both MINOCA and CABG-referred STEMI patients.

## 2. Materials and Methods

### 2.1. Study Population

A retrospective observational analysis was conducted using institutional ongoing STEMI registry from Cardiovascular Diseases Institute “George I.M. Georgescu” Iasi, Romania, which is affiliated to RO-STEMI (Romanian Registry for ST segment elevation myocardial infarction) [18]. The institutional STEMI registry encompasses all consecutive patients referred for primary PCI to our catheterization laboratory. Inclusion criteria were symptoms suggesting acute myocardial ischaemia of more than 30 minutes, time from onset of symptoms of less than 12 hours, and ST elevation of more than 0.1 mV in two or more ECG leads. Exclusion criteria were inability or refusal to provide informed consent, pregnancy, or declination to perform angiography.

Recently, we reported data from the same cohort of primary PCI patients dealing with in-hospital mortality and cardiogenic shock [19]. Our facility is a tertiary centre focused on coronary interventions (∼5000 procedures/year, and 1000 primary PCI/year), which provides a 24-hour primary PCI service to a population of 7,000,000 inhabitants, with a referral area that covers community hospitals from the northeastern part of the country.

The database was reviewed between 01 January 2017 and 01 January 2019, and 2035 consecutive patients who underwent coronary angiography for STEMI diagnosis listed as the indication for the procedure were included. Study protocol was approved by the ethical committee of Cardiovascular Diseases Institute “George I.M. Georgescu” Iasi. The analysis was conducted according to Declaration of Helsinki. No sex-based or racial-/ethnic-based differences were present.

### 2.2. Study Design, Methodology, and Data Collection

Demographic, clinical, biological, and paraclinical variables of our entire population have been described previously in detail [19].

STEMI patients receiving coronary angiography only were assigned to one of the following categories: (a) Takotsubo syndrome; (b) distal coronary embolisation; (c) myocardial infarction with nonobstructive coronary arteries (MINOCA) patients; (d) myocarditis; (e) CABG-referred patients (with severe coronary lesions); (f) normal coronary arteries (mostly diagnostic errors: early repolarization syndrome, Brugada syndrome, aortic dissection, subarachnoidal haemorrhage, and acute pulmonary embolism); and (g) others (patients refusing PCI and death prior to angioplasty).

Clinical data were obtained from patient's medical records. Assessed variables included demographic factors (age and sex), risk factors (diabetes mellitus, dyslipidemia, smoking history, arterial hypertension, and chronic kidney disease (eGFR <60 mL/min/m^2^), previous cardiovascular events/preexisting cardiovascular atherosclerotic disease, and cardiac (hs-cTnI and CK-MB) and inflammatory markers (C-reactive protein).

All consecutive patients presenting with STEMI diagnostic and referred for primary PCI were explored through a transthoracic echocardiographic protocol prior to cath lab admission. The swift echocardiographic examination was performed by two experienced sonographers, with the patient in the supine position. The protocol consisted of obtaining parasternal short and long axis and apical two- and four-chamber images. Tissue Doppler imaging was not performed, minimizing delays. End-diastolic and end-systolic left ventricular (LV) volumes and left ventricular ejection fraction (LVEF) were calculated, and wall motion abnormalities were recorded. LVEF was assessed using Simpson's biplane method.

### 2.3. Definitions of Pathological Entities Included in Analysis

Diagnosis of myocardial infarction was based on the third and later on the fourth universal definition of MI [20, 21] and included clinical evidence of acute myocardial ischaemia with detection of a rise and/or fall of cTn values, coexisting with symptoms, new ECG changes, and imaging/angiographic evidence attributable to an ischemic etiology.

- The diagnostic of Takotsubo cardiomyopathy was established according to the International Takotsubo Diagnostic Criteria (InterTAK Diagnostic Criteria) [22]. These include transient left ventricular dysfunction as apical ballooning or midventricular, basal, or focal wall motion abnormalities; emotional, physical, or neurologic disorders preceding the event, acting as triggers for Takotsubo syndrome; new ECG abnormalities; and elevated cardiac biomarkers (Table I from [22]).
- Distal coronary embolisation: presence of a thrombus defined as a noncalcified filling defect outlined on at least 3 sides by contrast media and the absence of coronary lesions (underlying the thrombus as well as throughout the coronary tree) [23], with a distal location of the occlusion and a relatively small area of myocardium at jeopardy [24, 25]. Differences between the two observers were resolved by consensus.
- Myocardial infarction with nonobstructive coronary arteries—MINOCA: as angiographic criterion for “nonobstructive coronary arteries,” we used the conventional cutoff of <50% stenosis, with a positive acute myocardial infarction diagnostic, consistent with contemporary angiographic guidelines [26].
- Myocarditis was diagnosed according to diagnostic criteria for clinically suspected myocarditis presented in the position statement of the European Society of Cardiology (Table 4 from [27]).
- CABG-referred patients (with severe coronary lesions)—in patients with complex CAD, the decision of surgical revascularisation was based on a heart team consensus. Heart team meetings consisted of minimum 3 physicians, including an interventional cardiologist, a cardiovascular surgeon, and a noninvasive cardiologist, usually the specialists on-call. Indications for CABG included significant left main disease, three-vessel disease or two-vessel disease (with significant involvement of the proximal left anterior descending coronary artery and either depressed left ventricular function or noninvasive evidence of ischaemia), or ventricular septal rupture/acute mitral regurgitation.
- Normal coronary arteries: this category was composed mainly of diagnostic errors (misinterpretation as STEMI yielding normal angiographic exam)—early repolarization syndrome, Brugada syndrome, aortic dissection, subarachnoidal haemorrhage, and acute pulmonary embolism [28].
- Other unusual situations—patient refusal and death before PCI—were also reported.

Procedural characteristics were assessed by the interventional cardiologist at the time of the PCI. At least two orthogonal angiographic projections were used to assess the coronary lesions, and they were evaluated according to the ACC/AHA classification [29]. All patients were treated following standard protocols recommended by guidelines.

### 2.4. Statistical Analysis

Data were statistically analyzed using the *χ*2 test and Fisher's exact test for comparing categorical variables, student's *t*-test for comparing group means, and also one-way ANOVA for continuous variables. A *p* value <0.05 was considered significant. Statistical analyses were conducted with IBM SPSS Statistics Version 20.0.

## 3. Results

From the entire population of 2035 patients with STEMI included in our registry, we identified 412 patients that received coronary angiography only and did not demand coronary angioplasty (either balloon or stent). These patients accounted for 20.2% of cath lab activations.

Baseline characteristics of the overall population, coronary angiography only group, and all seven subgroups are listed in Table 1.

Mean age of the population not receiving stent/balloon was 62.23 years (higher than the entire population, 60 years, *p*=0.23). There were 34% women in the same group (vs. 38.9% in all database, *p*=0.09). Only a quarter of the patients from the angio-only group was smokers (versus 67.1% in the entire population, *p*=0.05). Almost the same percentage of patients was diabetic (26.9% vs 27.4%, *p*=0.9), but only 54 patients (13%) had chronic kidney disease (vs. 19.4% from the entire database, *p*=0.1). However, a double percentage (40%) of patients from the angio-only group had severe LV dysfunction compared with 20% in the entire study group (*p*=0.04).

We assigned the 412 patients from the angio-only group into seven subgroups according to different pathologies (Table 1): (a) 8 patients with Takotsubo disease, (b) 68 patients with distal embolisation, (c) 70 patients with MINOCA, (d) 14 patients with myocarditis, (e) 165 patients referred to CABG, (f) 77 patients with normal coronary arteries, and (g) ten patients with other situations (patient refusal and death before PCI). Almost 75% of these angiographies was represented by patients referred to CABG, with MINOCA or with distal coronary embolisation, respectively.

To note, only 77 patients in two years of STEMI interventions (3.8% from all patients and 18.7% from angio-only group) implied a false-activation of cath lab team, yielding normal coronary arteries and subsequently diagnostic errors. In fact, this subgroup reported the youngest average age (55.7 years) and lower rates of comorbidities (13% with diabetes and 7.8% with CKD), but in a complex clinical context of either aortic dissection, subarachnoidal haemorrhage, acute pulmonary embolism, or Brugada syndrome.

40% of angio-only patients (*n* = 165 procedures) were referred to surgery due to a high complexity of atherosclerotic lesions (significant left main disease, three-vessel disease or two-vessel disease with significant involvement of the proximal left anterior descending coronary artery, and either depressed left ventricular function or noninvasive evidence of ischaemia), ventricular septal rupture, or acute mitral regurgitation in STEMI. They were older patients (mean age 66.72 years) with a higher burden of cardiovascular risk factors (one third had diabetes, two thirds had hypertension, 20% had CKD, and half of them had depressed LVEF). Almost the same high proportion of risk factors was observed in the distal embolisation group.

We performed univariate analysis assessing correlations between each pathological category with every cardiovascular risk factor recorded (Table 2).

No risk factor manifested correlations with Takotsubo syndrome patients (probably due to the small number of patients of the subgroup). However, the patients with distal coronary embolisation showed strong correlations with older age, smoking, hypertension, dyslipidemia, and lower LVEF. The MINOCA group also proved to have a solid correlation with smoking, diabetes, hypertension, and chronic kidney disease. However, the patient with hyperlipidemia had less chance of MINOCA in our study population. Smoking, hypertension, and dyslipidemia were correlated with the myocarditis group, while CABG-referred population was strongly associated with smoking, diabetes, dyslipidemia, hypertension, and LVEF <35%.

Moreover, the older patients were less likely to be referred for CABG, probably due to significantly more distal embolisations and normal coronary arteries. Surprisingly, the patients having diagnostic errors and STEMI misinterpretation with normal coronary arteries displayed strong correlations with all cardiovascular risk factors (Table 2).

## 4. Discussions

From our STEMI registry, we reported two-year consecutive patients (*n* = 2035) who required cath lab team activation. The main issue we focused on was the group of patients which received coronary angiography only (not followed by a stent/balloon angioplasty). We found that this group consisted of 20% of our patients (*n* = 412).

Probably, the most important question in organizing and auditing a cath lab and a STEMI network is as follows: if no stent/balloon was implanted, what was the procedure for? How could one evaluate the benefits of performing a coronary angiography (only) in a STEMI setting? [17].

From the interventional cardiologist point of view, being called at 2 o'clock in the night for a blank coronary angiography generates mixed feelings between enjoying that he/she does not need to undergo a complex coronary dilation and to be upset that he/she has been called in the middle of the night “for nothing.” On the other side, from the auditors point of view, the big dilemma is the correctness of including this type of patient in the STEMI network, thus activating a complex (and numerous) team and using expensive facilities and devices [30].

We identified smaller studies that defined “false-activation” and false-positive STEMI as absence of a clear culprit lesion on coronary angiography [31–33]. However, this definition excludes coronary distal embolisation or MINOCA, both being true MIs without a culprit lesion. In fact, one third of our angio-only group was made up of the above two entities (Table 1). Even if both entities require medical therapy “only,” it is obvious that coronary angiography was the key element for choosing the treatment between either dual antiplatelet therapy and high dose of statins (for MINOCA) or to chronic oral anticoagulation therapy (in distal coronary embolisation due to atrial fibrillation).

Another study stressed on the importance of defining “false-positive activation” and STEMI diagnosis: “*the frequency of false-positive cardiac catheterization laboratory activation for suspected STEMI is relatively common in community practice, depending on the definition of false-positive*” [34]. This observation encouraged us to stratify all the “angio-only” situations (accounting for 20% of our registry) into seven categories (Table 1).

Beside embolisation and MINOCA, we found that CABG-referred STEMI patients were the most numerous (*n* = 165, almost 40% from all 412 patients). Since our hospital has the cardiac surgery on-site, it is explicable that all complex cases with severe atherosclerotic unstable lesions or with mechanical complications were immediately referred to CABG. In addition, Takotsubo syndrome, myocarditis, or other severe pathologies accounted together for 8% from the angio-only group.

Finally, 77 patients from our registry had normal coronary arteries, due to diagnostic errors—early repolarization syndrome, Brugada syndrome, aortic dissection, subarachnoidal haemorrhage, or acute pulmonary embolism. We consider that this value (meaning 3.8 percent of the entire database) reflects the real number of false-STEMI activation. We also suggest that each STEMI department should record and report all the situations requiring angiography only, stratifying them as we proposed in the present study.

However, the results of the univariate analysis performed in our study also demand discussions. First, it seems reassuring that CABG-referred patients correlated well with most cardiovascular risk factors and with depressed LVEF, thus reflecting the complexity and severity of the coronary lesions from most of the patients. Second, we demonstrated that the distal embolisation group associated also with numerous risk factors, since they are probably connected with undiagnosed or neglected atrial fibrillation. In addition, since we defined the MINOCA group as having nonsignificant atherosclerotic stenosis, we also found a solid association between MINOCA and cardiovascular risk factors.

We expected the patients from the normal coronary arteries subgroup to show no correlation with our variables (since they have noncoronary pathologies), but in fact, almost all of them proved to be strongly associated with this group. Maybe, this is the very explanation for deciding to activate the cath lab team: a patient with atypical symptoms and abnormal EKG changes, but a patient with many cardiovascular risk factors “*convinces*” the doctors from the emergency department or from the cardiac intensive care unit to call the interventional cardiologist for an angio.

Finally, we acknowledge some limitations of our study. The proximity of the surgical clinic probably influenced the decision to refer some STEMI patients to CABG. It would be interesting to analyze a similar registry but in a tertiary centre without surgery. We also did not record those MINOCA patients who received a stent in a second procedure due to recurrent angina. In the same subgroup, we did not perform FFR or IVUS assessments due to lack of protocols in acute setting and operators' decisions. Also, there is a potential bias from unmeasured confounding factors not included in this analysis.

## 5. Conclusions

Our registry study reporting data from more than 2000 patients reflects a “real world” situation of the STEMI network and cath lab activations. STEMI network professionals often confront with coronary angiography only situations. We propose a classification according to the etiologies, and we suggest that the next STEMI guidelines should include audit recommendations and specific thresholds regarding angio-only STEMI patients, with a specific focus on MINOCA, CABG referrals, and distal embolisations. These measures will have a double impact: doctors will know in which category to allocate the patient, and financiers will have a clear perception about the usefulness of the investments.

## Figures and Tables

**Table 1:** Main characteristics of the entire study population and coronary angiography “only” categories.

Characteristics, patients No. (%)	Total population	Coronary angiography “only” patients	(a) Takotsubo	(b) Distal embolisation	(c) MINOCA	(d) Myocarditis	(e) CABG-referred patients	(f) Normal coronary arteries	(g) Others
	*n* = 2035 (100)	*n* = 412 (20.2)	*n* = 8 (2)	*n* = 68 (16.5)	*n* = 70 (17)	*n* = 14 (3.4)	*n* = 165 (40)	*n* = 77 (18.6)	*n* = 10 (2.5)
Mean age years, SD	60 ± 10.2	62.23 ± 114.3	67 ± 113.6	62.1 ± 113.5	63.5 ± 113.6	31.3 ± 18.3	66.72 ± 110.8	55.7 ± 114.9	70.7 ± 18.7
Age>70 y, *n* (%)	1145 (56.3)	125 (30.3)	4 (50)	18 (26.5)	21 (30)	0 (0)	65 (39.4)	12 (15.6)	5 (50)
Female gender, *n* (%)	792 (38.9)	140 (34)	5 (62.5)	24 (35.3)	25 (35.7)	1 (7.1)	50 (30.3)	29 (37.7)	6 (60)
Smoking, *n* (%)	1366 (67.1)	104 (25.2)	1 (12.5)	17 (25)	25 (35.7)	3 (21.4)	43 (26.1)	13 (16.9)	2 (20)
Diabetes mellitus, *n* (%)	558 (27.4)	111 (26.9)	0 (0)	19 (27.9)	19 (27.1)	0 (0)	62 (37.6)	10 (13)	1 (10)
Dyslipidemia, *n* (%)	1146 (56.3)	93 (22.6)	3 (37.5)	49 (72.1)	55 (78.5)	2 (15.4)	116 (70.6)	41 (53.3)	6 (75)
Hypertension, *n* (%)	1167 (57.3)	273 (66.3)	3 (37.5)	50 (73.5)	55 (78.6)	1 (7.1)	117 (70.9)	41 (53.2)	6 (60)
Chronic kidney disease, *n* (%)	394 (19.4)	54 (13.1)	3 (37.5)	9 (13.2)	3 (4.3)	0 (0)	32 (19.4)	6 (7.8)	1 (10)
LVEF<35%, *n* (%)	403 (19.8)	168 (40.8)	4 (50)	33 (48.5)	20 (28.6)	1 (7.1)	85 (51.5)	20 (26)	5 (50)

**Table 2:** Univariate analysis of selected study subgroups and cardiovascular risk factors.

	(a) Takotsubo (OR, 95% CI)	(b) Distal embolisation (OR, 95% CI)	(c) MINOCA (OR, 95% CI)	(d) Myocarditis (OR, 95% CI)	(e) CABG–referred patients (OR, 95% CI)	(f) Normal coronary arteries (OR, 95% CI)	(g) Others (OR, 95% CI)
Age >70	0.5 (0.11–2.26)	**3.14** ^*∗*^ **(1.25**–**6.37)**	1.13 (0.66–1.99)	∞ (1.74–∞)	**0.50** ^*∗*^ **(0.33**–**0.76)**	**1.25** ^*∗*^ **(1.01**–**1.56)**	0.33 (0.08–1.23)
Female gender	0.57 (0.13–2.54)	1.26 (0.75–2.18)	1.26 (0.76–2.14)	∞ (1.92–∞)	0.74 (0.54–1.02)	**2.66** ^*∗*^ **(1.4**–**4.78)**	0.39 (0.09–1.39)
Smoking	0.98 (0.19–4.17)	**3.19** ^*∗*^ **(1.91**–**5.32)**	**3.01** ^*∗*^ **(1.82**–**4.9)**	**21.17** ^*∗*^ **(2.76**–**62.19)**	**4.15** ^*∗*^ **(2.93**–**5.86)**	**2.76** ^*∗*^ **(1.72**–**4.41)**	1.08 (0.27–3.92)
Diabetes mellitus	2.45 (0.42–62.55)	1.15 (0.67–2.07)	**6.9** ^*∗*^ **(4.2**–**11.4)**	1.39 (0.42–6.44)	**1.12** ^*∗*^ **(1.07**–**1.68)**	**1.9** ^*∗*^ **(1.07**–**3.60)**	1.49 (0.36–10.84)
Dyslipidemia	2.32 (0.55–12.09)	**5.1** ^*∗*^ **(2.96**–**8.84)**	**0.38** ^*∗*^ **(0.20** –**0.66)**	**18.73** ^*∗*^ **(2.44**–**14.3)**	**5.5** ^*∗*^ **(3.9**–**7.9)**	**1.25** ^*∗*^ **(1.2**–**2.1)**	0.96 (0.23–3.47)
Hypertension	∞ (1.86–∞)	**2.83** ^*∗*^ **(1.65**–**4.86)**	**3.02** ^*∗*^ **(1.77**–**5.16)**	**∞** ^*∗*^ **(3.64**–**∞)**	**1.9** ^*∗*^ **(1.3**–**2.64)**	**7.74** ^*∗*^ **(3.96**–**15.14)**	**9.68** ^*∗*^ **(1.24**–**17.8)**
Chronic kidney disease	0.36 (0.08–1.86)	1.41 (0.73–3.09)	**5.06** ^*∗*^ **(1.58**–**16.19)**	∞ (0.73–∞)	0.90 (0.61–1.37)	**2.66** ^*∗*^ **(1.15**–**6.17)**	1.76 (0.32–43.96)
LVEF <35%	0.31 (0.07–1.41)	**3.42** ^*∗*^ **(1.92**–**5.12)**	0.79 (0.47–1.37)	3.69 (0.73–89.99)	**2.62** ^*∗*^ **(1.9**–**3.63)**	0.90 (0.54–1.55)	0.32 (0.09–1.18)

^*∗*^Statistically significant, *p* < 0.05 at least.

## Data Availability

The data used to support the findings of this study are available from the corresponding author upon request.
